# Bilateral diabetic Charcot neuroarthropathy of the knee in a young woman with diabetes suspected of Wolfram-like syndrome

**DOI:** 10.1007/s13340-026-00895-5

**Published:** 2026-04-22

**Authors:** Teruo Jojima, Toshie Iijima, Masaaki Sagara, Kanako Suda, Masahiro Saito, Dai Tanuma, Shintaro Sakurai, Takuya Tomaru, Yuji Yamaguchi, Isao Usui, Hiroshi Taneichi, Yoshimasa Aso

**Affiliations:** 1 Department of Endocrinology and Metabolism, Dokkyo Medical University, Mibu, Tochigi 321-0293 Japan; 2 Department of Department of Orthopedic Surgery, Dokkyo Medical University, Mibu, Tochigi Japan

**Keywords:** Charcot neuroarthropathy, Knee: diabetic neuropathy, Wolfram-like syndrome, WFS1

## Abstract

We described a very rare case of bilateral diabetic Charcot neuroarthropathy of the knee in a young woman who may have Wolfram-like syndrome. The onset of diabetes occurred at the age of 11. At the age of 26, she consulted an orthopedic surgeon for pain and swelling in the left knee with no history of trauma. Pain and swelling of the left knee got worse despite treatment with knee orthosis. On physical examination, her left knee was hot and swollen with pain, while her right knee seemed to be intact. An X-ray of the left knee revealed a horizontal discontinuity of the cortical bone at the medial plate and bone defects on the medial tibial articular surface, while an X-ray of the right knee also revealed bone fragmentation in the femoral lateral condyle. We made a diagnosis of bilateral diabetic Charcot knee joints. The patient had a novel mutation, which is a missense heterozygous mutation of 650 tyrosine to cysteine in WFS1; thus, a diagnosis of Wolfram-like syndrome (WFLS) was suspected.

## Introduction

Charcot neuroarthropathy is a progressive, non-infectious destruction of bone and joints (osteoarthropathy) in peripheral neuropathy, which is well recognized in people with long-standing diabetes [1–3]. Evidence of loss of deep sensation and proprioception due to diabetic polyneuropathy is associated with poor joint protection. The proprioceptive deficit and the motor instability could be the cause of undetected microtrauma [1–3]. Diabetic sympathetic neuropathy would lead to increased blood flow and bone resorption. This combination could lead to a pathological fracture of the foot [1–3].

Diabetic Charcot arthropathy typically affects the joints of the forefoot and ankle. Charcot neuroarthropathy affecting the knee in diabetes is rare; thus, the diagnosis is delayed, resulting in detrimental outcomes [4]. Moreover, bilateral involvement is extremely rare [5, 6]. Here we described a very rare case of bilateral diabetic Charcot neuroarthropathy of the knee in a young woman with diabetes due possibly to Wolfram-like syndrome.

## Case presentation

At the age of 11, she was diagnosed with diabetes and immediately received insulin therapy. At the age of 20, she discontinued her diabetes treatment after starting a job. At the age of 26, she consulted an orthopedic surgeon for persistent pain and swelling in the left knee with no history of trauma. Despite treatment with knee orthosis, pain and swelling of the left knee got worse. Simultaneously, since her HbA1c was elevated at 14%, she resumed insulin therapy. And then she was admitted to our department of our hospital for glycemic control and evaluation of diabetic complications. She had a family history of type 2 diabetes for her father, mother, and maternal grandmother. Her mother had insulin treatment after being diagnosed with type 2 diabetes.

On physical examination, her body mass index was 18.1 kg/m^2^,, and her left knee was hot and swollen with spontaneous pain, while her right knee seemed to be intact. Her ankle reflexes were absent, and she had reduced sensation to pain, touch, and temperature. Pretibial and pedal edema was found on both her legs. An ulcer also was found on the right hallux. Her grip strength was 10.3 kg on the right and 12.6 kg on the left.

She had not only sensorimotor neuropathy but also autonomic neuropathy, including orthostatic hypotension. Diabetic proliferative retinopathy was found in both her eyes.

Laboratory findings showed that serum alkaline phosphatase (ALP) was markedly increased to 1386 U/L. She had anemia (8.8 g/dL of hemoglobin, 26.8% of hematocrit). An estimated glomerular filtration rate (eGFR) was 147.7 mL/min/1.73m^2^, while the urinary albumin creatinine ratio (UACR) was 667 mg/gCr. Several bone resorption markers were highly elevated [urinary cross-linked N-telopeptide of type I collagen (NTx), 192.1 nmol bone collagen equivalents/mmol; urinary deoxypyridinoline, 11.0 nmol/mmol Cr; serum tartrate-resistant acid phosphatase 5b, 484 mU/dL]. Dual-energy X-ray absorptiometry showed an extremely low bone mineral density with a mean femur T-score of − 3.3 SD.

**Fig. 1:**
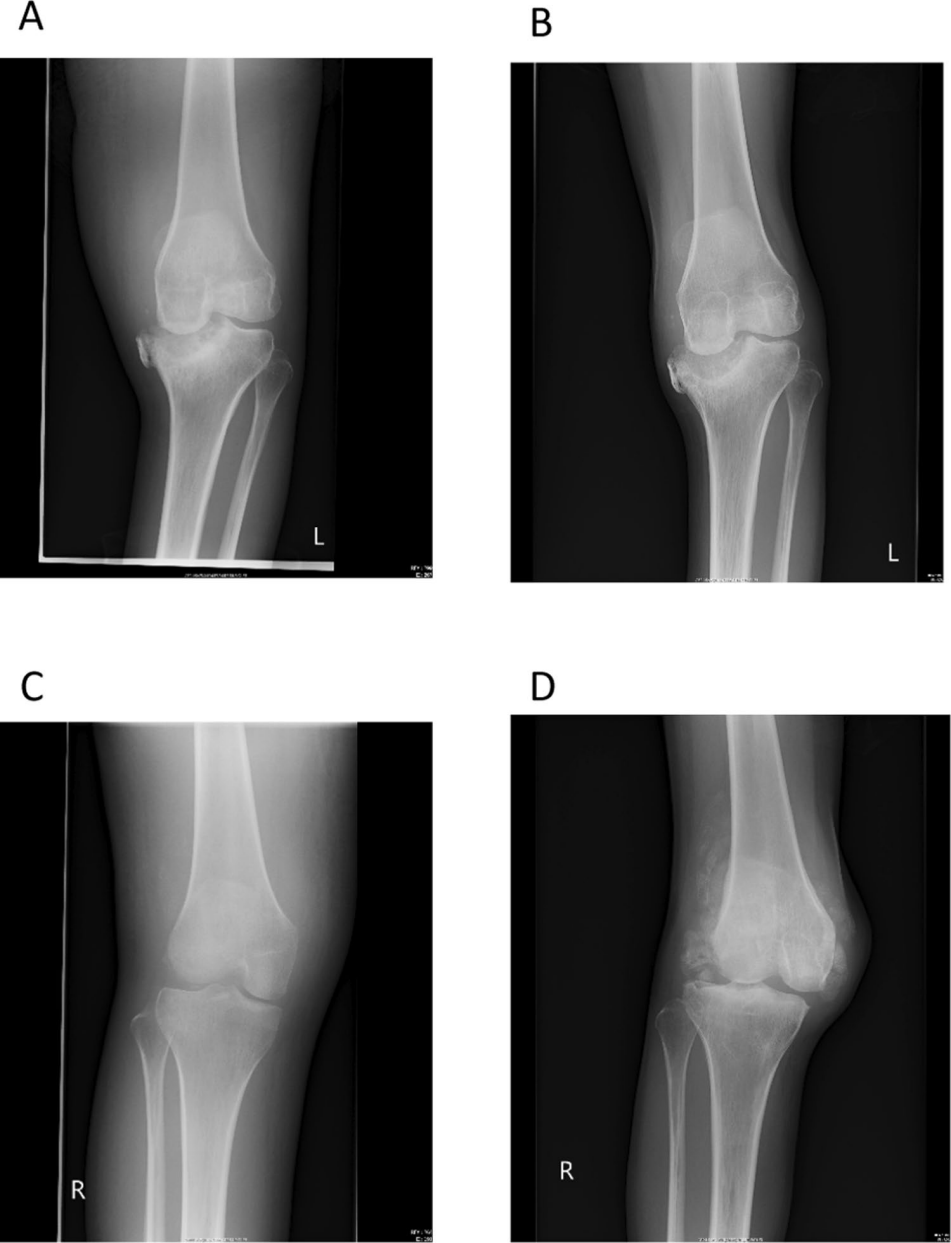
X-rays of the left (**A**, **B**) and right knee (**C**, **D**) at the time of hospitalization and one year later

**Fig. 2:**
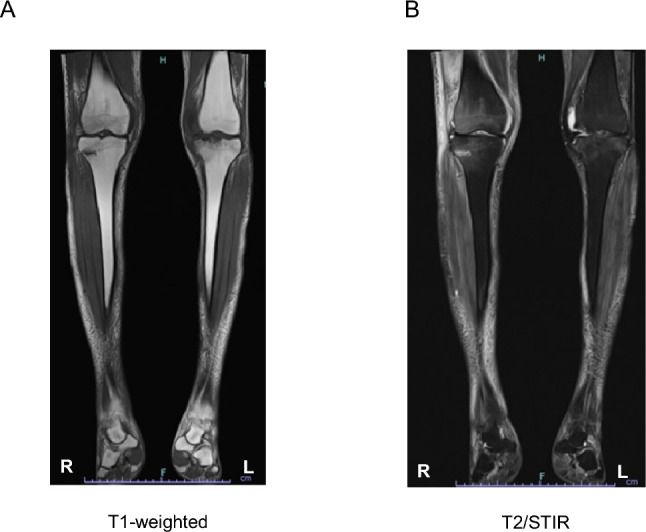
T1-weighted (**A**) and T2/STIR (**B**) MRI images of the right and left knees

On admission, X-rays of the left knee showed depression of both the left and right sides of the medial tibial plateau and a bone defect the medial tibial articular surface (Fig. 1A), while X-rays of the right knee also revealed bone fragmentation in the femoral lateral condyle (Fig. 1C). We found that bone destruction of the lateral femoral condyle with collapse of the articular surface and lateral tibial eminence with bone resorption of the left tibia was accompanied by the knee effusion (Fig. 1A). On T1-weighted MRI images, low signal is observed mainly at the lateral condyle of the right femur and the proximal lateral malleolus of the tibia (Fig. 2A), while T2/STIR images show high signal in these areas (Fig. 2B). The MRI scans also shows compression of the left tibial plateau, accompanied by soft tissue signals in the same area, and joint effusion is also observed (Fig. 2A and B).

We then performed fine-needle aspiration of knee arthrocentesis fluid. There were no detected bacteria in the culture of knee arthrocentesis fluid. Cultures of the synovial fluid had no bacteria. Charcot neuroarthropathy is a systemic disease characterized by progressive arthropathy and bone destruction, which is usually closely related to diabetes and neurosyphilis. However, Charcot neuroarthropathy can be seen in spinal fistula, hereditary neuropathy, spina bifida, and syringomyelia as well as in diabetes [9]. Therefore, to investigate the cause of this disease other than diabetes, serum tests and an MRI of the vertebrae were performed, but no other underlying conditions were found. Taken together, we made a diagnosis of bilateral diabetic Charcot neuroarthropathy of the knee.

Because most commonly, diabetic Charcot neuroarthropathy affects the ankle joints and the forefoot, we carefully examined with an ankle X-ray whether the lesion was on the ankle joints and the forefoot. No Charcot neuroarthropathy was found in the ankle joints or the forefoot.

One year later, X-rays of the left knee showed lysis of the tibial metaphysis with periosteal reaction and irregular sclerotic borders (a varus deformity of the tibia) (Fig. 1B). The destruction of the right knee joint progressed rapidly, because one year later, an X-Ray of the right knee revealed a proximal medial tibial fracture with subluxation, bony destruction with fragmentation, periarticular bone formation, and proximal fracture of the fibula (Fig. 1D).

During this hospitalization, since the HbA1c level was 14.4%, indicating extremely poor blood glucose control, basal bolus insulin therapy was initiated and administered at 40 units per day (8 units of insulin glargine and 10, 12, and 10 units of insulin aspart before breakfast, lunch, and dinner, respectively). The C-peptide index (CPI), which indicates endogenous insulin secretory capacity, showed a value of 0.54, suggesting an impairment of insulin secretion.

We suspected the existence of monogenic diabetes, because her diabetes developed at age 11 and no antibodies to islet antigens, such as GAD, IA-2, and ZnT8, were detected. We first examine the presence of mitochondrial DNA mutation 3242 (A > G); however, no mutation was found. Next, using next-generation sequencing, we performed sequencing analysis of the coding exons and adjacent intron regions of several genes that cause monogenic diabetes, including HNF4A, GCK, HNF1A, PDX1, HNF1B, NEUROD1, INS, ABCC8, KCNJ11, WFS1, and INSR. We found a novel mutation, which is a missense heterozygous mutation of 650 Tyr (tyrosine) to Cys (cysteine) in WFS1. According to the guideline of the American College of Medical Genetics and Genomics (ACMG)/CMG sequence variant classification, this mutation indicated Class 3, which means variant of uncertain significance (VUS). We underwent thorough examination in both the otorhinolaryngology and ophthalmology departments; she had no hearing impairment (childhood-onset) or optic atrophy.

The orthopedic surgeon planned total knee arthroplasty (TKA) of both knees while waiting for this disease to transition from the acute phase of Charcot neuroarthropathy to the chronic phase, allowing the enhanced bone resorption to settle down. Two years later, her Charcot neuroarthropathy has reached a chronic phase, and TKA is being planned. However, due to poor blood glucose control (HbA1c 8–9%), TKA surgery is currently on hold. To improve glycemic control, insulin doses are being gradually increased (16 units of insulin glargine and 10, 22, and 16 units of insulin aspart before breakfast, lunch, and dinner, respectively).

## Discussion

Diabetic Charcot neuroarthropathy is a rare but serious consequence of diabetic neuropathy, affecting between 0.8 and 8% of people with diabetes [1, 7]. Since diabetic Charcot neuroarthropathy typically affects the joints of the forefoot and ankle, Charcot neuroarthropathy of the knee in people with diabetes is extremely rare. Diabetic Charcot neuroarthropathy of the knee is a rare clinical entity, as only 0.45% of all diabetic patients are likely to suffer from this condition [8]. A previous report also demonstrated that there were only three cases of Charcot knee out of 281 cases of diabetic Charcot neuroarthropathy of the foot [4]. Diabetic Charcot knee is thus rare on one side and even rarer when it is on both sides [5, 6].The diagnosis is often made late due to the unspecific early presentation of brutal inflammatory joint pain, which can also be misdiagnosed as a common fracture, infectious and rheumatic arthritis, deep venous thrombosis, algoneurodystrophy, or erysipelas [9]. The delay in its diagnosis results in detrimental outcomes, such as joint destruction and pathological bone fracture. Evidence of loss of deep sensation and proprioception due to diabetic polyneuropathy is associated with poor joint protection and undetected microtrauma [1–3]. That observation will be the starting point for the diagnosis. Prompt diagnosis and conservative management are appropriate in the acute phase to maintain joint structural integrity.

As far as we are aware, there are only 16 cases of diabetic Charcot neuroarthropathy of the knee—including ours—reported globally (Table 1). As shown in Table 1, it is interesting to note that 88% (14 instances of 16) were female with type 1 diabetes [4–6, 10–18]. Although it is well known that there is no gender difference in the incidence of diabetic Charcot neuroarthropathy of the ankle [1–3], there is clearly a gender difference in the onset of diabetic Charcot knee. Women are overwhelmingly more likely to be affected by this disease compared to men. In particular, we speculate that diabetic Charcot knee occurs specifically in long-term poorly controlled women with type 1 diabetes who usually are very thin (Table 1).

**Table 1:** Current reports on the clinical features of patients with diabetic Charcot neuroarthropathy of the knee

	Gender	Age	BMI	Diabetes	Duration of diabetes	HbA1c (%)	Knee joint	Treatment	References
1	Female	21	–	Type 1	17	(poor)	right	Total keen arthroplasty (TKA)	Endocrine [10]; 49:863–864
2	Female	21	–	Type 1	> 18	(poor)	right	Bone transplantation	The Knee [11];20:58–62
3	Female	48	–	Type 1	–	–	right	Conservative → TKA	Rheumatol Adv Pract. [12]; 29:2(1):rky004
4	Male	44	–	Type 1	36	–	right	Conservative non-weight bearing	Case Rep Orthop. [13]; 2018:9,301,496.
5	Female	46	–	Type 1	32	–	left	Above-knee total contact cast	Case Rep Orthop. [13]; 2018:9,301,496
6	Female	27	–	Type 1	12	(poor)	right	TKA, bisphosphonate	Diabet Med [14]; 19: 338–341
7	Female	25	–	Type 1	9	(poor)	right	Zimmer cast, non-weight bearing	Joint Bone Spine. [15];82(6):474–5.
8	Female	22	16.0	Type 1	> 16	(poor)	left	Non-weight bearing	Isr Med Assoc J. [16]; 22(10):650–651.
9	Female	69	–	Type 2	–	(poor)	right	TKA	Lancet [17]; 372; 1854
10	Female	40	–	Type 1	–	–	bilateral	TKA (both)	Case Rep Orthop. [6]; 2016:3,204,813
11	Female	61	–	Type 2	12	(poor)	bilateral	TKA (right), knee arthrodesis (left)	Arch Phys Med Rehabil. [5]; 78(7):780–2.
12	Female	25	–	Type 1	10	11.6	–	–	Diabetes Care [4]; 37; e129-30
13	Female	26	–	Type 1	16	9.8	–	–	Diabetes Care [4]; 37; e129-30
14	Female	30	–	Type 1	13	9.1	–	–	Diabetes Care [4]; 37; e129-30
15	Male	38	26.3	Type 2	5	5.7	left	TKA	Cureus [18]; 14 (8):e28163
Our case	Female	26	18.1	s/o Wolfram-like syndrome	15	14.4	bilateral	Conservative, no-weight bearing → Scheduled TKA (both)	–

Mechanisms responsible for the higher incidence rate of diabetic Charcot knee in women than in men remain to be determined. One possible explanation is that muscle weakness, including of the quadriceps, may contribute to higher incident rate of diabetic Charcot knee in women than in men. Women tend to have weaker muscle strength in areas such as the quadriceps compared to men, which can increase the burden on the knees [19]. In particular, women with type 1 diabetes tend to be thin and exhibit a decrease in lower limb muscle strength. Knee osteoarthritis is more prevalent in women and is linked to early muscular changes and muscle weakness, which facilitate disease progression [20]. Another explanation is that a wider pelvis of women compared with men makes it easy for the knee to experience twisting and stress, increasing the burden on the joint in the long term [21]. The entire leg is easily subjected to a lateral thrust force, which puts strain on the knees [21]. For the above reasons, women are more susceptible to diabetic Charcot arthropathy of the knee than men. A third possible explanation is that sex hormone imbalance is a contributing factor to severe bone loss and susceptibility to Charcot neuroarthropathy. In diabetic Charcot neuroarthropathy disease itself, regardless of gender, bone resorption is markedly increased [1]; however, in our case of a thin young woman, an imbalance in sex hormones, particularly estrogen deficiency, might contribute to the development of Charcot neuroarthropathy through increased bone resorption. A fourth possible explanation is the association of the muscle-bone interaction with the development of diabetic Charcot neuroarthropathy. As anatomically nearby organs, skeletal muscle and bone work together to control motor function through mechanical interactions [22]. Unfortunately, in our case, we did not measure skeletal muscle mass. However, muscle strength was evaluated using grip strength. Her grip strength was 10.3 kg on the right and 12.6 kg on the left. Thus, a marked decrease in muscle strength was observed, suggesting the presence of sarcopenia. The relationship between sarcopenia and osteoporosis may be derived from the bone-muscle interactions [22]. Thus, the bone-muscle interactions may have contributed collaboratively to the onset of diabetic Charcot neuroarthropathy.

Interestingly, our patient had a missense heterozygous mutation of 650 Tyr to Cys in exon 8 of WFS1. The WFS1 gene has eight exons, with exon 8 as the largest exon being the most significant, and mutations are concentrated in exon 8 [23]. The WFS1 gene mutation in our patient was also located in exon 8. The WFS1 gene codes an 890-amino acid transmembrane protein. WFS1 protein has nine transmembrane domains and localizes in the endoplasmic reticulum (ER) membrane. A mutation in exon 8 induces ER stress, leading to protein misfolding or proteostasis, leading to cell death and neurodegeneration [23]. We suspected a diagnosis of Wolfram-like syndrome (WFLS), but not Wolfram syndrome (WS), because WFLS is a described autosomal dominant disorder with phenotypic similarities to autosomal recessive WS regarding optic atrophy, hearing impairment, and diabetes mellitus [24, 25]. The cause of her diabetes onset in adolescence is possibly due to be WFLS. WFLS has generally a milder phenotype in comparison to WS. However, since she had no hearing impairment (childhood-onset) or optic atrophy [24, 25], there may be a low possibility that we make a diagnosis of her illness as WFLS. Moreover, according to the guideline of the American College of Medical Genetics and Genomics and the Association for Molecular Pathology. (ACMG/CMG) sequence variant classification [26], this mutation indicated Class 3, which means variant of uncertain significance (VUS). Despite the increasing number of studies on WFLS, a clear phenotypic description of this disease is not yet available, and there are no known genotype–phenotype correlations [24, 25]. The mutations in the WFS1 gene are highly heterogeneous, and, thus far, this novel mutation of our patient does not present a clinically significant appearance. Thus, the diagnosis of WFSL is merely a speculation in our case.

The development of Charcot neuroarthropathy involves not only diabetic sensorimotor neuropathy but also autonomic neuropathy (1). Although autonomic neuropathy and sensorimotor neuropathy are seen in patients with Wolfram syndrome [27], Wolfram-like syndrome and Charcot neuroarthropathy are unlikely to be related in our case. This mutation of WFS1 is classified as a variant of uncertain significance (VUS) without functional validation. In actuality our case had no hearing impairment (childhood-onset) or optic atrophy. We concluded that the development of Charcot knee in this patient is most plausibly explained by long-standing, poorly controlled diabetes with severe sensorimotor and autonomic neuropathy (Table 1).

In conclusion, we reported a very rare case of bilateral diabetic Charcot neuroarthropathy of the knee in a young woman who may have Wolfram-like syndrome. Bilateral involvement of knee Charcot is more exceptional than unilateral. Charcot neuroarthropathy of the knee is rare and possibly underdiagnosed as a complication of diabetes, leading to irreversible damage to the knee joints. If you see a long-standing diabetic female patient with microvascular complications complaining of progressive knee pain and instability in the absence of trauma and with rapidly destructive arthropathy on X-ray, clinicians should promptly pay attention to this diagnosis. The existence of one-sided Charcot knee should raise doubts about the existence of the opposite side.

## Data Availability

The data that support the findings of this report are available from the corresponding author on reasonable request.

## References

[1] Rajbhandari SM, Jenkins RC, Davies C, Tesfaye S. Charcot neuroarthropathy in diabetes mellitus. Diabetologia. 2002;45(8):1085–96. 12189438 10.1007/s00125-002-0885-7

[2] Wukich DK, Frykberg RG, Kavarthapu V. Charcot neuroarthropathy in persons with diabetes: it’s time for a paradigm shift in our thinking. Diabetes Metab Res Rev. 2024;40(3):e3754. 38069459 10.1002/dmrr.3754

[3] Bell DSH, Jerkins T. Diabetic Charcot neuroarthropathy: a threat to both limb and life. Diabetes Obes Metab. 2025;27(1):35–9. 39382008 10.1111/dom.15994

[4] Illgner U, van Netten J, Droste C, Postema K, Meiners T, Wetz HH. Diabetic Charcot neuroarthropathy of the knee: conservative treatment options as alternatives to surgery: case reports of three patients. Diabetes Care. 2014;37(6):e129–30. 24855166 10.2337/dc13-3045

[5] Fullerton BD, Browngoehl LA. Total knee arthroplasty in a patient with bilateral Charcot knees. Arch Phys Med Rehabil. 1997;78(7):780–2. 9228885 10.1016/s0003-9993(97)90090-3

[6] Goetti P, Gallusser N, Borens O. Bilateral diabetic knee neuroarthropathy in a forty-year-old patient. Case Rep Orthop. 2016;2016:3204813. 27668112 10.1155/2016/3204813 PMC5030418

[7] Gouveri E, Papanas N. Charcot osteoarthropathy in diabetes: a brief review with an emphasis on clinical practice. World J Diabetes. 2011;2:59–65. 21691556 10.4239/wjd.v2.i5.59 PMC3116009

[8] Babazadeh S, Stoney JD, Lim K, Choong PF. Arthroplasty of a Charcot knee. Orthop Rev (Pavia). 2010;2(2):e17. 21808708 10.4081/or.2010.e17 PMC3143972

[9] Lu V, Zhang J, Thahir A, Zhou A, Krkovic M. Charcot knee: presentation, diagnosis, management—a scoping review. Clin Rheumatol. 2021;40(11):4445–56. 34031760 10.1007/s10067-021-05775-8 PMC8143744

[10] Allo Miguel G, García Fernández E, Hawkins Carranza F. Diabetic Charcot neuroarthropathy of the knee in a patient with type-1 diabetes mellitus. Endocrine. 2015;49(3):863–4. 25351369 10.1007/s12020-014-0466-9

[11] Date H, Hayakawa K, Yamada H. Allograft bone transplantation for neuropathic arthropathy of the knee associated with type 1 diabetes mellitus. Knee. 2013;20(1):58–62. 22763341 10.1016/j.knee.2012.06.002

[12] Daniel A, Alegre C, Judas F, Fonseca F. Diabetic Charcot neuroarthropathy of the knee: a rare clinical entity. Rheumatol Adv Pract. 2018;2(1):rky004. 31431953 10.1093/rap/rky004 PMC6649931

[13] Patel A, Saini AK, Edmonds ME, Kavarthapu V. Diabetic neuropathic arthropathy of the knee: two case reports and a review of the literature. Case Rep Orthop. 2018;2018:9301496. 29610694 10.1155/2018/9301496 PMC5828462

[14] Lambert AP, Close CF. Charcot neuroarthropathy of the knee in Type 1 diabetes: treatment with total knee arthroplasty. Diabet Med. 2002;19(4):338–41. 11943008 10.1046/j.1464-5491.2002.00704.x

[15] Jacquemin C, Petitdidier N, Davidowicz K, Guignard S, Ajzenberg C, Chevalier X. Diabetic neuroarthropathy: report of a rare case of a Charcot knee. Joint Bone Spine. 2015;82(6):474–5. 26184540 10.1016/j.jbspin.2014.11.008

[16] Giryes S, Militianu D, Balbir-Gurman A. Neuropathic arthropathy of a knee joint: a case report. Isr Med Assoc J. 2020;22(10):650–1. 33070492

[17] Kong MF, Nayyar V, Jogia R, Berrington R, Jackson S. A swollen knee: and the father of hysteria. Lancet. 2008;372(9652):1854. 19027485 10.1016/S0140-6736(08)61760-2

[18] Kojima C, Himeno T, Akao M, Kamiya H, Nakamura J. Multifocal neuroarthropathy of the knee and foot induced by physical training of the lower extremities in a patient with Latent Autoimmune Diabetes in Adults. Cureus. 2022;14(8):e28163. 36148208 10.7759/cureus.28163 PMC9482758

[19] Behan FP, Maden-Wilkinson TM, Pain MTG, Folland JP. Sex differences in muscle morphology of the knee flexors and knee extensors. PLoS ONE. 2018;13(1):e0190903. 29360834 10.1371/journal.pone.0190903 PMC5779647

[20] O’Connor MI. Sex differences in osteoarthritis of the hip and knee. J Am Acad Orthop Surg. 2007;15(Suppl 1):S22–5. 17766785

[21] Weidow J, Mars I, Kärrholm J. Medial and lateral osteoarthritis of the knee is related to variations of hip and pelvic anatomy. Osteoarthritis Cartilage. 2005;13(6):471–7. 15922181 10.1016/j.joca.2005.01.009

[22] Kaji H. Bone-muscle interactions. Osteoporos Sarcopenia. 2025;11(2 Suppl):32–9. 40718354 10.1016/j.afos.2025.04.001 PMC12288928

[23] Kõks S. Genomics of wolfram syndrome 1 (WFS1). Biomolecules. 2023;13(9):1346. 37759745 10.3390/biom13091346 PMC10527379

[24] Grenier J, Meunier I, Daien V, Baudoin C, Halloy F, Bocquet B, et al. WFS1 in optic neuropathies: mutation findings in nonsyndromic optic atrophy and assessment of clinical severity. Ophthalmology. 2016;123(9):1989–98. 27395765 10.1016/j.ophtha.2016.05.036

[25] de Muijnck C, Brink JBT, Bergen AA, Boon CJF, van Genderen MM. Delineating Wolfram-like syndrome: a systematic review and discussion of the WFS1-associated disease spectrum. Surv Ophthalmol. 2023;68(4):641–54. 36764396 10.1016/j.survophthal.2023.01.012

[26] Richards S, Aziz N, Bale S, Bick D, Das S, Gastier-Foster J, et al. Standards and guidelines for the interpretation of sequence variants: a joint consensus recommendation of the American college of medical genetics and genomics and the association for molecular pathology. Genet Med. 2015;17(5):405–24. 25741868 10.1038/gim.2015.30 PMC4544753

[27] Urano F. Wolfram syndrome: diagnosis, management, and treatment. Curr Diab Rep. 2016;16(1):6. 26742931 10.1007/s11892-015-0702-6 PMC4705145

